# Parallelized Field-Programmable Gate Array Data Processing for High-Throughput Pulsed-Radar Systems

**DOI:** 10.3390/s25010239

**Published:** 2025-01-03

**Authors:** Aaron D. Pitcher, Mihail Georgiev, Natalia K. Nikolova, Nicola Nicolici

**Affiliations:** 1 Electromagnetic Vision (EMVi) Research Laboratory, McMaster University, Hamilton, ON L8S 4L8, Canada; georgims@mcmaster.ca (M.G.); talia@mcmaster.ca (N.K.N.); 2 Computer-Aided Design and Test (CADT) Research Group, McMaster University, Hamilton, ON L8S 4L8, Canada; nicolici@mcmaster.ca

**Keywords:** concealed weapon detection, field-programmable gate array, equivalent-time sampling, subsampling, ultra-wideband measurement techniques, ultra-wideband radar

## Abstract

A parallelized field-programmable gate array (FPGA) architecture is proposed to realize an ultra-fast, compact, and low-cost dual-channel ultra-wideband (UWB) pulsed-radar system. This approach resolves the main shortcoming of current FPGA-based radars, namely their low processing throughput, which leads to a significant loss of data provided by the radar receiver. The architecture is integrated with an in-house UWB pulsed radar operating at a sampling rate of 20 gigasamples per second (GSa/s). It is demonstrated that the FPGA data-processing speed matches that of the radar output, thus eliminating data loss. The radar system achieves a remarkable speed of over 9000 waveforms per second on each channel. The proposed architecture is scalable to accommodate higher sampling rates and various waveform periods. It is also multi-functional since the FPGA controls and synchronizes two transmitters and a dual-channel receiver, performs signal reconstruction on both channels simultaneously, and carries out user-defined averaging, trace windowing, and interference suppression for improving the receiver’s signal-to-noise ratio. We also investigate the throughput rate while offloading radar data onto an external device through an Ethernet link. Since the radar data rate significantly exceeds the Ethernet link capacity, we show how the FPGA-based averaging and windowing functions are leveraged to reduce the amount of offloaded data while fully utilizing the radar output.

## 1. Introduction

The advent of ultra-wideband (UWB) technology has enabled a multitude of emerging sensing and imaging applications. It operates in various frequency bands between 100 MHz and 10.6 GHz [1]. Its main advantages are non-ionizing radiation, low-power emission spread over large bandwidths, and good penetration [2], especially in the low-GHz spectrum. Emerging applications include medical sensing and diagnostics [3,4,5], security and surveillance [6,7,8,9,10], through-the-wall imaging [11,12,13,14,15,16,17], and ground penetrating radar [18,19,20,21,22].

Sensing and imaging applications of UWB technology demand time-domain measurements with sampling rates on the order of tens of gigasamples per second (GSa/s), along with versatility, affordability, and compactness. Versatility greatly simplifies the system development for a new application, whereas the low cost and small size/weight enable mobile and large-scale network deployment. The proof-of-concept development of a new custom UWB radar application often employs bench-top instruments (e.g., high-speed oscilloscopes and waveform generators) as they are, by design, versatile, i.e., capable of generating and measuring a variety of radar waveforms. They are also highly accurate. However, they are large, heavy, and expensive, and advancing the prototype to a practical application often hinges on developing a custom UWB radar system. Here, we propose an accurate FPGA-based low-cost, compact, and versatile UWB receiver.

To date, a few compact UWB receiver prototypes have been reported in the literature, but they are application-specific [3,4,5,9,10,11,12,13,14,15,16,17,18,19]. Also, important challenges have not been addressed. First, high sampling rates demand fast processing and high data-transfer rates that match the sampling speed and prevent data loss so that the radar capabilities are fully utilized. Second, fast data offloading to a computer is needed for application-specific signal processing and data storage. Finally, time-domain receivers are prone to noise and interference due to their large input bandwidths. While bench-top oscilloscopes [23,24,25,26,27] and off-the-shelf high-speed analog-to-digital converters (ADCs) [28,29,30] can meet the required sampling rates, their data transfer to an external device creates a bottleneck. Since they do not support custom signal processing, data offloading to an external computing platform is needed, which creates a major impediment. Also, the cost of bench-top oscilloscopes and GSa/s ADC chips is prohibitive when a low-cost solution is desired.

The reported compact custom UWB receivers [3,4,5,9,10,11,12,13,14,15,16,17,18,19] employ various architectures. Here, we are interested in direct equivalent-time (ET) sampling receivers, i.e., those without down-conversion, that use lower ADC sampling rates, leading to simple data interfaces and reduced cost. It has been shown in [3,9,10,11,12,13,14,15,16,18,19,31] that ET sampling can be implemented on field-programmable gate arrays (FPGAs), but only a few of these sources [11,12,13,14,15,16,31] describe aspects of the FPGA firmware architecture that performs the radar control and the ET waveform processing in the receivers. From the reported throughput performance, it is apparent that current architectures suffer from significant data loss in the data-processing pipeline. One reason is the time needed to adjust and stabilize (or settle) the clocks controlling the delays applied to the ADC sampling [11,12]. This time is very significant when a phase-locked loop (PLL) delay-control network is employed since it requires approximately a microsecond [32,33] to phase-lock on the clock signal and create the sampling delay. In the case of PLLs, the settling time is greater than the ADC’s sampling period and is of the same order of magnitude as the pulse repetition period (or pulse cycle). This results in less than 50% of the pulse cycles being utilized, thereby reducing the waveforms per second at the radar output [12]. An alternative is to employ a programmable delay chip (PDC) [14,15], which has much shorter settling times (on the order of picoseconds [34]). PDCs offer an advantage because the settling time is orders of magnitude smaller than the ADC’s sampling period. Therefore, there is no loss of pulses within the received pulse train. The FPGA-based radar receiver proposed here employs a PDC to eliminate the loss of pulse cycles due to clock settling and to highlight the importance of parallelizing the firmware architecture for fully utilizing the pulse train.

Another factor contributing to data loss in prior FPGA processing architectures is the use of a single memory bank to perform the waveform reconstruction, the waveform averaging, and the offloading [11,12,13,15,16,31]. This sequential approach creates a data-throughput bottleneck. Memory bank offloading (reading) takes time comparable to waveform reconstruction (implemented as memory bank writing), slowing down processing by a factor of 2 if reading and writing occur at the same rate. In the absence of buffering, only reading or writing can occur at one time, leading to data loss within the FPGA pipeline. But FPGAs support multiple memory banks, enabling buffering so that reconstruction can be carried out in parallel with offloading, thereby completely eliminating data loss.

Here, we propose a parallelized FPGA architecture designed to control a generic receiver, which functions as a highly accurate, high-speed oscilloscope [10] and supports a wide range of UWB radar applications. The parallelized FPGA architecture maximizes the data throughput and efficiency, achieving 98.2% utilization of the received pulse train. The design features simultaneous data collection from two ADC channels while optimizing parallelization for the per-channel computation. The proposed parallel processing minimizes data loss and maximizes data throughput. This implementation leverages eight memory banks, of which four are used for waveform reconstruction and interference monitoring (two per receiver channel) and another four for user-defined averaging (two per receiver channel).

The proposed architecture supports real-time radar applications requiring ultra-fast measurements, such as tracking moving objects. Capturing thousands of measurements per second enables advanced detection and identification tools, including those using statistical signal processing or machine learning, which require large datasets. This work does not focus on detection and imaging (radar signal processing) algorithms. For example, in our concealed weapon detection application, novel statistical algorithms are implemented on external computing platforms for research and development purposes, emphasizing the system’s need for high data throughput and efficiency. Although introduced in the context of this application, the system is versatile and adaptable to various use cases. It can integrate with any transmitter module (e.g., pulse or continuous-wave generators) that supports synchronization, adding versatility.

The FPGA architecture leverages the multi-bank design to perform signal-processing tasks crucial for signal quality assurance: (i) user-defined waveform averaging to improve signal-to-noise ratio (SNR), and (ii) interference monitoring and suppression. To the best of our knowledge, FPGA-based processing for interference suppression has not previously been implemented. This is critically important since UWB radars share frequencies with cellular, Wi-Fi, and Bluetooth signals. These additional tasks do not affect the data throughput rate.

Finally, we introduce a systematic method, along with metrics, for evaluating data loss in the FPGA signal-processing chain, as well as that in the data offloading from the UWB radar system to an external device (e.g., a computer). To the best of our knowledge, these rates have either not been investigated or not reported in published work. The throughput of image frames per second (FPS) was reported in [11,12,14], but this metric included image reconstruction. A waveform refresh rate at the FPGA output was reported in [31], but the data loss or efficiency of the FPGA processing is unknown. We demonstrate the proposed method and metrics with the new parallelized FPGA architecture on the dual-channel UWB receiver when this receiver is connected to an external computer through an Ethernet link. A 1 gigabit per second Ethernet link is used because it is the fastest interface on the employed FPGA board [35]. The radar output of over 9000 waveforms per second (Wfm/s), generating 6.4 gigabits per second (Gbps), greatly exceeds the capacity of the Ethernet link. Therefore, we show how the FPGA-based functionality is leveraged to reduce the offloaded data while fully utilizing the radar output.

This paper is organized as follows. Section 2 discusses the requirements for the UWB radar system. Section 3 describes the proposed FPGA architecture. The results are discussed in Section 4, including throughput analysis and measurements involving moving people. Conclusions are provided in Section 5.

## 2. UWB Radar System Requirements

### 2.1. The Intended Application

The detection method proposed in [6,7,8] provides a non-imaging solution for unattended and unobtrusive radar surveillance to detect weapons concealed under clothing or in bags. The need for such surveillance arises from the limited efficacy of current screening technologies (e.g., walk-through detectors [36], X-ray and millimeter-wave full-body scanners [37], portable scanners [38]), all of which require trained security personnel in proximity to the inspected person, who must comply with instructions and remain still. This results in low screening throughput and precludes around-the-clock automated surveillance.

To be effective, the method proposed in [8] requires a low-cost, compact radar system, which enables discrete deployment, preferably multiple units for wide-area coverage and various viewing angles of an object. Additionally, it benefits from polarization diversity. For this reason, our radar system is equipped with two transmitting (Tx) and two receiving (Rx) antennas. The antennas are linearly polarized, acquiring four back-scattered responses: VV, HH, VH, and HV. Here, H stands for horizontal and V stands for vertical; the first letter denotes reception, whereas the second denotes transmission.

### 2.2. Frequency Bandwidth and Pulse Excitation Requirements

Non-imaging detection methods distinguish an object of interest from the radar background and clutter by analyzing the early-time response and late-time response of the back-scattered radar signals or by extracting features based on estimated radar-cross section and polarization ratios [6,7,8,39,40,41,42]. In particular, the method proposed in [8] relies on the resonant signatures of the various weapons of interest (handguns, knives, grenades, or explosive vests), which are typically found in the range of approximately 400 MHz to 5 GHz [6,39]. Our UWB radar system is designed to operate from 500 MHz to 5 GHz.

**Figure 1:**
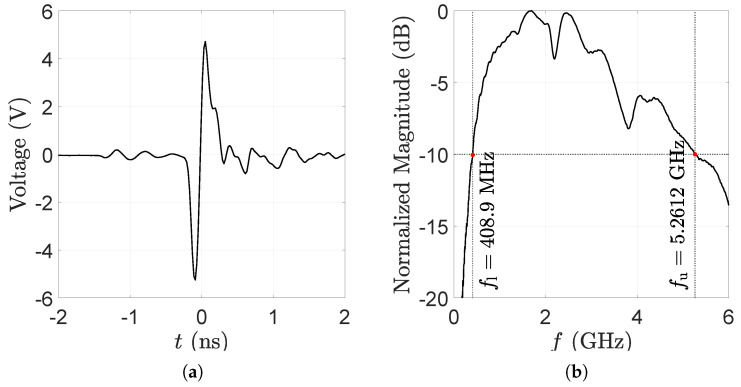
The UWB radar system’s monocycle-like pulse generated by a picosecond pulse generator [43]: (**a**) temporal plot, (**b**) spectral plot indicating the lower $\left(f_{l}\right)$ and upper ($f_{u}$) bounds with red dots for the −10 dB bandwidth.

The excitation is provided by a picosecond pulse generator, which produces a highly stable monocycle-like pulse [43]. The measured temporal and spectral plots of the pulse are shown in Figure 1. The −10 dB bandwidth covers the spectrum from 500 MHz to 5 GHz.

### 2.3. Timing Requirements

The measurement speed must ensure multiple waveform acquisitions while the object remains within the radar’s field of view. The signal-processing algorithms that issue the threat/non-threat decision and identify the threats must also perform in real time; however, they run on an external computer and are not discussed here. Here, we focus on data acquisition speed and signal preprocessing on the FPGA-based system. The main constraint is the time available while the radar interrogates a moving person.

**Figure 2:**
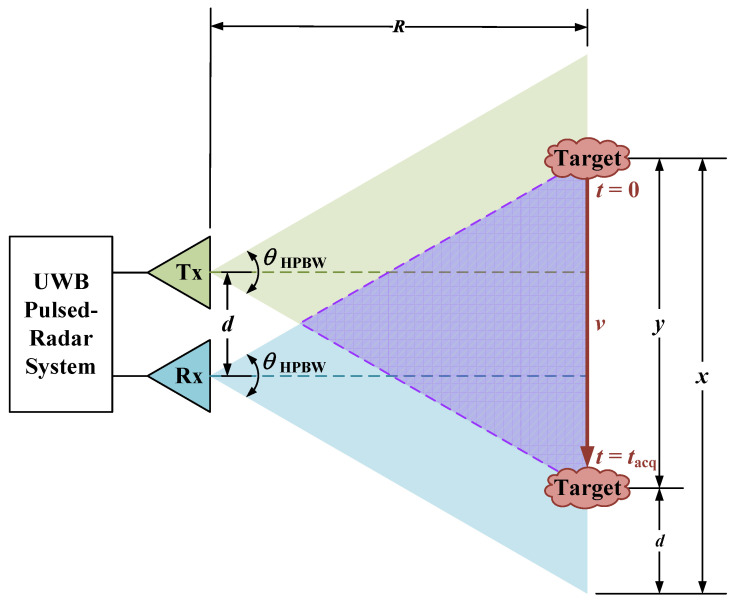
Data acquisition window of target.

The average velocity *v* of a person, walking or running, ranges from 0.75 m/s to 3.25 m/s [44]. Figure 2 shows an example arrangement for the intended application, where the Tx and Rx antennas are placed apart at a distance of d=0.5m. The object is at a distance *R*, which is smaller than the radar’s unambiguous range, and moving perpendicularly to the radar’s field of view, resulting in the shortest observation time. The maximum unambiguous range for a monostatic radar configuration is $R_{\max}=cT_{P}/2$, where *c* is the speed of light and $T_{P}$ is the inverse of the pulse repetition frequency (PRF).

The purple-shaded region, outlined by dashed lines, is where the object is within both the Tx and Rx antennas’ field of view. Using the UWB antenna half-power beamwidth (HPBW) of $\theta_{\mathrm{HPBW}}=51^{\circ }$ [45], the distance traveled by the object while in this region is

(1) $$y=x-d=2R\tan(\theta_{\mathrm{HPBW}}/2)-d.$$

The acquisition time is $t_{\mathrm{acq}}=y/v$. The shortest acquisition time arises at the shortest range distance R≈1 m, for which $138\;\mathrm{ms}≲t_{\mathrm{acq}}≲605\;\mathrm{ms}$ if 0.75m/s≲v≲3.25m/s. Within this short observation time, it is desirable to acquire more than a hundred measurements in each of the four available radar responses (VV, HH, VH, and HV). The range R≥1 m ensures operation beyond the reactive near-field zone of the antennas.

With the above requirement in mind, the chosen PRF for the monocycle-like pulse of each transmitter is $f_{\mathrm{PRF}}=1$ MHz ($T_{P}=1$ μs). The two transmitters are triggered on the rising edges of their respective synchronized 1 MHz clock signals, which have a 50% duty cycle and are mutually shifted by 0.5 μs. Thus, the effective PRF of the system is $T_{P,\mathrm{eff}}=0.5$ μs. Note that the unambiguous radar range with this $T_{P,\mathrm{eff}}$ is ∼75 m, which exceeds the targeted concealed weapon detection (CWD) application range (∼1 m to 10 m).

The two pulse generators trigger about 276,000 pulses for the shortest observation time, 138 ms. On the other hand, as explained later, each of the two ET receiver channels sub-samples 110 waveforms to reconstruct a single, fully sampled waveform. We emphasize that each fully sampled waveform contains two back-scattered responses (co- and cross-polarized), spaced in time by 0.5 μs. Thus, the radar system, with its two Tx and two Rx antennas, can provide over 5000 back-scattered responses within the shortest observation time. Such data abundance enables highly effective statistical detection and identification methods. This remarkable speed is conditional upon FPGA processing and offloading to the computer in real time without data loss.

### 2.4. Implementation of the Radar System

**Figure 3:**
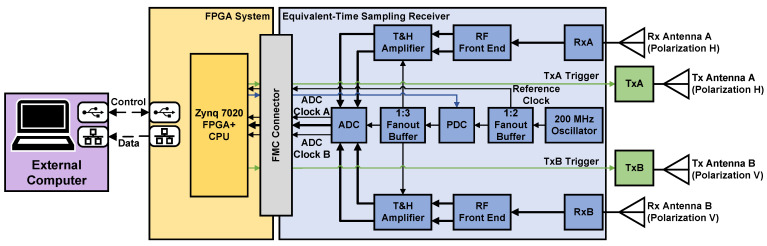
High-level block diagram of the UWB pulsed-radar system. RxA and RxB are the two ETSR Rx input channels. TxA and TxB are the two Tx modules.

Figure 3 presents a high-level block diagram of the proposed dual-channel UWB pulsed-radar system. Table 1 summarizes the key radar parameters. The system simultaneously receives on two Rx channels (RxA and RxB). With two sequentially excited Tx modules (TxA and TxB) and antennas, it performs full polarimetric measurement with four responses. It consists of two identical transmitter modules (the picosecond pulse generator described in [43]), the output of which is shown in Figure 1. The custom dual-channel equivalent-time sampling receiver (ETSR) features an input bandwidth of 6.1 GHz at the −10 dB level and an ET sampling rate of 20 GSa/s [10]. The dual-channel ETSR receiver interfaces with a low-cost FPGA board [35] through two 200 MHz low-voltage differential signal (LVDS) data buses (one per ADC channel) across the FPGA mezzanine card (FMC) connector. The common off-the-shelf FPGA board controls the entire system. We emphasize that the proposed radar system is coherent, i.e., the ETSR is precisely synchronized with the employed Tx modules. Finally, the data are offloaded to the external computer for target feature extraction and classification. The radar has a programmable PRF, here set to $f_{\mathrm{PRF}}$.

Figure 3 shows the key ETSR hardware components and their clock distribution network, which are essential for understanding the FPGA design. A detailed description of the ETSR is provided in [10]. A 200 MHz oscillator [46] generates the *reference clock*, distributed to the FPGA and PDC via a 1:2 fan-out buffer [47]. The FPGA uses this clock for internal logic and to synchronize the Tx modules with the waveform reconstruction. The 1 MHz TxA and TxB triggers (see Figure 3) are derived from this reference clock within the FPGA and sent to the Tx modules. The PDC [34], discussed later, introduces delays to shift the ADC sampling points relative to the oscillator. A 1:3 fan-out buffer [48] distributes the delayed clock to the ADC and the two track-and-hold (T&H) amplifiers. Precise clock timing is crucial to account for internal delays in the T&H amplifiers and ADC [9], ensuring that the Rx signals are synchronized and sampled during the T&H amplifier’s hold stage. The ADC digitizes samples into 16-bit values, sending them over the LVDS data buses along with an ADC clock (see *ADC clocks A and B* in Figure 3). These clocks, synchronized with the PDC output, are a delayed version of the reference clock.

Next, we describe how the PDC is leveraged to perform ET sampling, the efficiency of which critically depends on the FPGA firmware architecture. The 20 GSa/s sampling rate requires an ET sampling interval Δt=50 ps. The ET waveform reconstruction interleaves *K* sub-sampled waveforms (K=110), each containing *N* samples (N=200) acquired by the onboard 200 MHz ADC [49]. The *k*-th sub-sampled waveform of a signal x(t) is

(2) $$x_{k}\left[n\right]=x\left(\tau_{k}+nT_{\mathrm{ADC}}\right),\;n=0,\ldots ,N-1$$

where $\tau_{k}$ (k=0,…,K−1) is the delay determining its start and $T_{\mathrm{ADC}}=5$ ns is the ADC’s sampling period. The delay $\tau_{k}$, which time-shifts the *k*-th sub-sampled waveform, is realized by the PDC, which is a cascaded network of delay circuits. Each delay circuit (referred to as a *tap*) is enabled or disabled by the FPGA. The 10 tap delays of the PDC are represented by the delay vector

(3) $$d={\left[4610,2300,1150,575,290,145,70,35,15,10\right]}^{T}\;,$$

in ps. The overall delay is $\tau_{k}=c_{k}^{T}d$, where $c_{k}\;\in \;{\left\{0,1\right\}}^{10}$ is a sequence of tap-control bits. For example, if $c_{k}\;=\;{\left[0,0,0,0,0,0,0,1,1,0\right]}^{T}$, then $\tau_{k}=50$ ps.

If the delays were equispaced, i.e., if $\Delta t=\tau_{k+1}-\tau_{k}=50$ ps for all *k*, exactly K=100 sub-sampled waveforms $x_{k}\left[n\right]$ are needed to reconstruct x(t), since $K=T_{\mathrm{ADC}}/\Delta t$. However, it is evident from (3) that the PDC cannot provide equispaced delays. For this reason, K=110 sub-sampled waveforms are used to provide a temporal overlap between the waveform samples $\left\{x_{100}\left[n\right]\cdots x_{109}\left[n\right]\right\}$ and $\left\{x_{0}\left[n+1\right]\cdots x_{9}\left[n+1\right]\right\}$. The PDC delays are optimally configured to provide tap-delay increments close to 50 ps, covering a tap-delay range from $\tau_{0}=0$ ps to $\tau_{109}=5450$ ps.

It is worth mentioning that the actual PDC tap delays may differ significantly from the tabulated values in (3) [10,50]. A calibration procedure has been developed to extract the true delay values [50]. This delay correction, along with interpolation to an equispaced 50 ps timebase (carried out on the external computer), results in an accurate uniformly sampled reconstructed signal with a temporal length of 1 μs.

**Figure 4:**
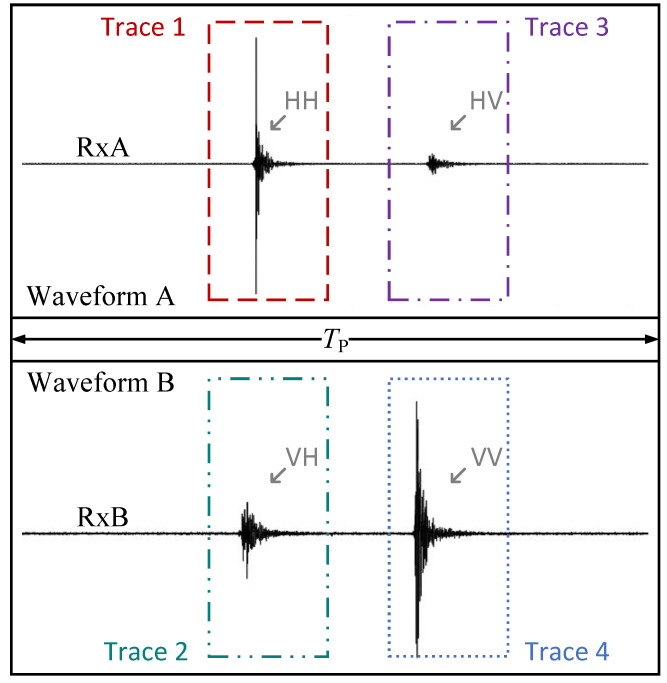
Visualization of the terms *waveform* and *trace* in relation to the four radar responses (VV, HH, VH, and HV). The plots are derived from actual measurements of a scattering object.

Hereafter, we adopt the following terminology for the reconstructed signals. The term *waveform* refers to a full 1 μs single-channel reconstruction due to reception from one Rx antenna. The dual-channel radar receiver’s simultaneous processing reconstructs two such waveforms. Further, a waveform contains two *responses* (HH and HV, or VH and VV), triggered 0.5 μs apart. Thus, a response is 0.5 μs long. Finally, the term *trace* refers to a user-defined portion of a response. Typically, the trace spans a time interval where the user expects to receive a back-scattered signal based on the radar range and/or the expected distance to the target. Thus, a trace has a length ≤0.5 μs. As shown later, the reduced trace size can significantly alleviate the burden of data offloading and effectively eliminate data loss. Figure 4 provides a visualization of this terminology.

### 2.5. Data Offloading Constraints

The speed requirement for data offloading is dictated by the speed at which data are generated by the dual-channel ETSR, whose dual-channel 200 MHz 16-bit ADC output data rate is 6.4 Gbps, or 800 MB/s. This data stream is then processed in the FPGA to produce the overall radar throughput, which is measured in terms of Wfm/s. In our case, the measurement time required to obtain a full waveform on each channel is $T_{\mathrm{Wfm}}=KT_{P}=110$ μs. Thus, ideally (no data loss), the waveform generation rate on each channel is $\mathrm{INT}(T_{\mathrm{Wfm}}^{-1})=9090$ Wfm/s, where INT(x) rounds *x* down to an integer. A single waveform reconstructed in the FPGA contains NK=200×110= 22,000 samples (with 16-bit samples), or 44 kB of data. Provided the FPGA preprocessing keeps up with the ADC in reconstructing the two output waveforms simultaneously, the radar output data rate is 2×44 kB × 9090 Wfm/s, or 799.92 MB/s, closely matching the ADC’s data rate.

The proposed FPGA board features a 1 Gbps (125 MB/s) Ethernet link, which we use for data offloading. Our receiver far exceeds the data rate of the widely available Ethernet link. Thus, we perform real-time signal preprocessing, such as interference monitoring, averaging, and trace windowing, to reduce the amount of offloaded data, achieving nearly 100% data utilization over this interface. Higher-throughput solutions would enable us to offload more raw data, but this would be of little utility in our envisioned application, which targets the employment of low-cost solutions.

## 3. Proposed FPGA System Design

**Figure 5:**
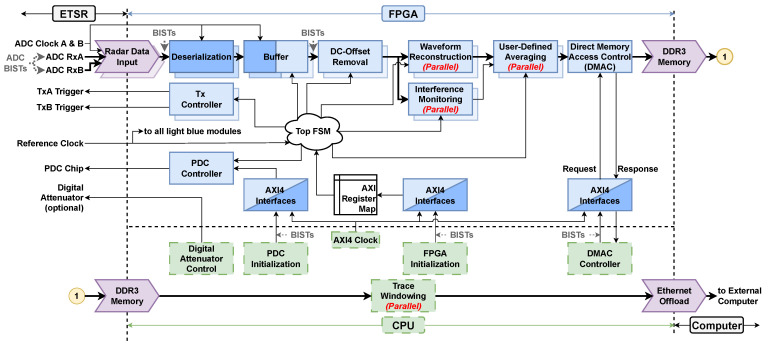
A high-level diagram of the data pipeline for waveform reconstruction and preprocessing on the FPGA and CPU SoC. The data pipeline is interrupted at the circle labeled 1 to wrap the image on the page. The blue blocks outlined by solid lines indicate processing occurring within the FPGA. The double-stacked blue blocks indicate simultaneous processing on two channels. The blocks labeled “Parallel” indicate where parallel processing is implemented. The green blocks outlined by dashed lines indicate processing on the CPU SoC. The purple chevron blocks represent interfaces for data transfer from/to the indicated source/destination. If a block is colored in two shades, it corresponds to a process involving clock-domain crossings (CDCs).

Our design utilizes a Zynq 7020 system-on-a-chip (SoC) [51], containing an Artix-7 FPGA and a dual-core ARM Cortex-A9 central processing unit (CPU) on a pre-built FPGA board [35]. Figure 5 presents the proposed firmware architecture, which leverages the FPGA’s high speed to perform real-time (RT) waveform reconstruction and embedded signal-preprocessing tasks. The preprocessing conditions the waveform before submitting it to the external computer for further processing. The dual-channel ADC data are simultaneously collected on both channels, while optimal parallelization is proposed for per-channel preprocessing. Here, we use the term “simultaneous processing” to refer to the concurrent data collection due to the dual-channel ADC (indicated by the stacked blue blocks in Figure 5). Meanwhile, the terms “parallel processing” or “parallelization” refer only to the optimal parallel operations in each of the two channels (indicated by the “parallel” labels in Figure 5), thus enabling the loss-free high throughput of data.

The FPGA implementation is written using hardware description languages, specifically *System Verilog*, *Verilog*, and *VHDL*, in AMD’s Vivado Design Suite [52]. The custom CPU codebase uses the *C*-based Software Development Kit [53] provided with Vivado [52].

With reference to Figure 5, the data flows from the ETSR ADC (top-left corner) through the FPGA data pipeline. Then, it is passed to the shared off-chip DDR3 memory (top-right corner) and the CPU (see circle 1). The CPU performs *trace windowing*, which reads the external DDR3 memory data and breaks the waveforms into traces. Then, the traces are offloaded by the CPU to the external computer through the 1 Gbps Ethernet link. As shown in the CPU section in Figure 5, the other CPU functions are: (i) setting the attenuation level in the ETSR radio frequency (RF) front end (*digital attenuator control*), (ii) start-up configuration of the PDC and the FPGA (*PDC initialization* and *FPGA initialization*), and (iii) requesting the last dual-channel reconstructed waveforms from the direct memory access control (DMAC) (*DMAC controller*).

We next focus on the FPGA section in Figure 5. The *top FSM* (finite state machine) provides master-control sequences within the FPGA. These are initialized by a register map provided by the CPU. The ADC output data stream must first be deserialized. The *deserialization* module receives the ADC samples in a serialized format and converts each of them into a 16-bit parallel bit stream for the *buffer*. The *buffer* (one per channel) queues the 16-bit samples of the *k*-th sub-sampled waveform. The *DC-offset removal* module removes the mean (DC offset) from the sub-sampled waveform (a parasitic effect created by the ETSR circuitry). The sub-sampled waveform is then passed to both the *waveform reconstruction* and *interference-monitoring* modules, which are the first two parallel processing modules in the proposed architecture. The reconstructed waveform is then passed to the third parallel processing module, the *user-defined averaging* module, if no electromagnetic interference (EMI) is detected. If EMI corrupts the waveform, it is discarded. This concludes waveform preprocessing within the FPGA. The waveform is then passed to the DMAC, where it is stored in the DDR3 memory at an address allocated by the CPU. As indicated by the double blue blocks in Figure 5, each module in the preprocessing chain is duplicated for simultaneous processing on both the RxA and RxB channels.

The key FPGA features that significantly enhance the per-channel data throughput through parallelization are explained next.

### 3.1. FPGA Synchronization

The proposed FPGA architecture operates in an asynchronous multi-clock system. First, the radar system has an external 200 MHz precision *reference clock* provided by the ETSR (refer to Figure 3). This is the same reference clock shown in Figure 5. All FPGA preprocessing must be synchronized with this clock (see the light-blue modules in Figure 5). Second, a 100 MHz advanced extensible interface (AXI) clock (generated within the FPGA) synchronizes the commands between the CPU and the FPGA on the SoC through the AXI4 Interfaces (see the three interfaces in Figure 5). Third, there are two 200 MHz ADC clocks (one per channel, labeled *ADC Clock A* and *ADC Clock B* in Figure 3 and Figure 5) provided by the ETSR ADC. These ADC clocks provide the timing reference for deserializing and buffering the ADC outputs into 16-bit parallel bit streams. We consider all four clocks to be mutually asynchronous, including the ADC clocks, which originate from the reference clock but pass through the PDC, introducing variable delays.

As a result, the FPGA architecture must accommodate domains synchronized by their respective clocks. These are referred to as *clock domains*, of which our architecture has four (reference clock, AXI clock, and two ADC clocks). When data are passed between clock domains, the metastability created by the clock-domain crossing (CDC) must be addressed [54,55]. A metastable state, referred to as metastability, occurs when the output of a flip-flop within the FPGA is unpredictable. At all CDCs, this is accomplished with asynchronous first-in first-out (FIFO) buffers [56], configured to use one clock reference for writing and another for reading. Provided the FIFO’s status flags are monitored within their respective clock domains (which they are here), an error-free CDC is achievable. Here, CDCs occur in the two *buffer* modules (see Figure 5), which are implemented as FIFOs capable of holding a minimum of *N* samples. Here, the FIFO depth is 512 samples. When the FIFO *writes*, filling the *buffer*, it is synchronized with the ADC clock. But when it *reads*, emptying the *buffer*, it is synchronized with the reference clock. The FIFO CDC implementation was verified using built-in self-test (BIST) patterns, e.g., deterministic synthetic binary signals generated by either the ADC (referred to as test patterns in [49]) or custom-built pattern generators embedded within the FPGA processing (see the BIST labels in Figure 5). These BIST patterns are used to emulate hardware and synthesize data buses to validate FPGA functionality in operational conditions. After verifying the BIST patterns through simulation and hardware, it was determined that no error correction and detection were needed. Similarly, the *AXI4 interfaces* use FIFOs, which can buffer 16 CPU commands. These FIFOs *write* to the AXI clock and *read* from the reference clock. Likewise, these CDCs were also verified using BIST patterns through FPGA simulations of the AXI interconnect and CPU commanding on the hardware, e.g., writing and then reading back known values to registers within the FPGA.

**Figure 6:**
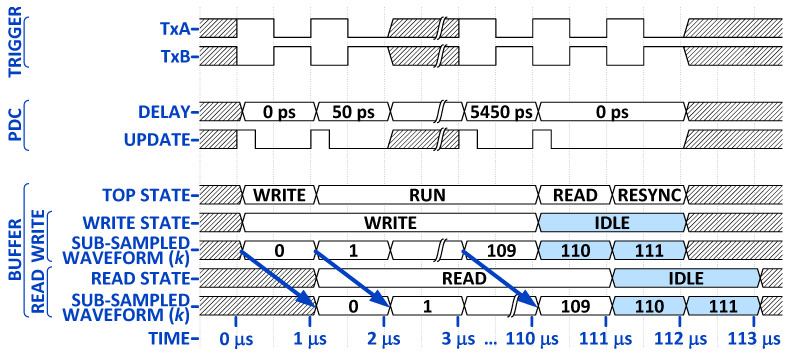
FPGA synchronization diagram for a single waveform reconstruction. The shaded regions are where the process repeats. The blue arrows indicate the data transfer of the *k*-th sub-sampled waveform through the *buffer*. The shaded 110 and 111 cells are two additional sub-sampled waveforms that cannot be received due to synchronization issues.

The synchronization of the two *buffers* with the *PDC controller* and the *Tx controller* is illustrated in Figure 6. All transitions are synchronized with the rising edges of the 200 MHz reference clock, which is not shown due to its short temporal extent. The TxA and TxB 1 MHz triggers are clock-divided from the reference clock, and they trigger a pulse transmission on the rising edge. A $180^{\circ }$ phase shift is applied to the TxB trigger to stagger the two transmitted pulses in time by 0.5 μs. A PDC delay setting is invoked on the rising edge of the TxA trigger, and the setting depends on the sub-sampled waveform index *k*, after which it resets to 0 ps (see the third row in Figure 6). Each new delay setting is invoked by the PDC update sequence, which is a latch signal generated by the FPGA (see the fourth row in Figure 6). The two *buffers* follow identical timing sequences, and only one is shown in rows five to nine. At the start of waveform reconstruction, the buffer’s *top state* is a write-only state (WRITE), during which the buffer accepts the first sub-sampled waveform (k=0), filling it with *N* samples. Then, it transitions to a RUN state, where it performs *write* and *read* operations for the sub-sampled waveforms k=1,…,K−2, and a *write* operation for the last sub-sampled waveforms k=K−1=109. The FIFO remains filled around *N* samples as the write and read clocks operate at the same rate (200 MHz) but with different phases. Finally, it transitions to a read-only state (READ), emptying the last sub-sampled waveform (*N* samples) until the FIFO empty flag is raised. CDC sample counters are used on both clock domains to ensure that the NK samples required for waveform reconstruction are passed through the FIFO. The buffer then enters a re-synchronization state (RESYNC). The blue arrows in Figure 6 represent the *k*-th sub-sampled waveform data transfer through the *buffer*. We can observe that two additional sub-sampled waveforms (shaded and labeled as 110 and 111 in rows seven and nine) are actually generated by the ADC while the *buffer* is in the READ and RESYNC top states when it cannot *write*. Since only 110 out of 112 waveforms are actually used, the data-throughput efficiency is 98.2% before the waveform reconstruction occurs.

Although the re-synchronization state causes minor data loss, it is necessary. In one period $T_{P}=1$ μs, the ADC provides N=200 samples for each sub-sampled waveform. But the ET sampling scheme requires a delay $\tau_{k}$ of the ADC clock (controlled by the PDC) for each (*k*-th) sub-sampled waveform, thus pushing its end into the next 1 μs period. For example, the sampling of the last 109th waveform is delayed by $\tau_{109}=5\;450$ ps, effectively extending the 1 μs waveform period to an ET waveform extent of 1005.45 ns. To synchronize the acquisition of the next set of 110 waveforms with the Tx triggers, the system needs to skip *writing* one waveform period. It also must wait for the *buffer* to offload (or *read*) the last sub-sampled waveform.

### 3.2. FPGA Waveform Reconstruction

The *waveform reconstruction* process in each channel (see Figure 5) interleaves the *K* sub-sampled waveforms, described by (2), into a single fully sampled waveform:

(4) $$x\left[k+nK\right]=x_{k}\left[n\right]=x\left(\tau_{k}+nT_{\mathrm{ADC}}\right)\;.$$

**Figure 7:**
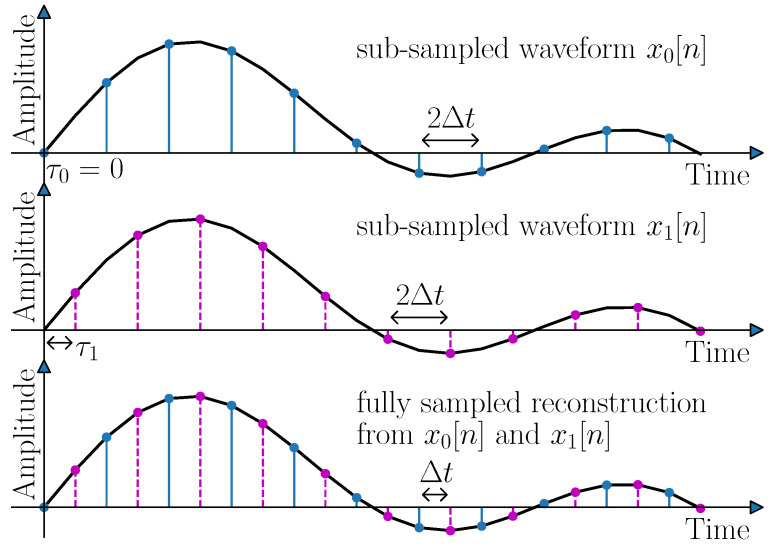
Illustration of waveform reconstruction by interleaving two sub-sampled waveforms.

Figure 7 illustrates the interleaving of K=2 sub-sampled waveforms to produce a waveform with an ET sampling step of Δt.

**Figure 8:**
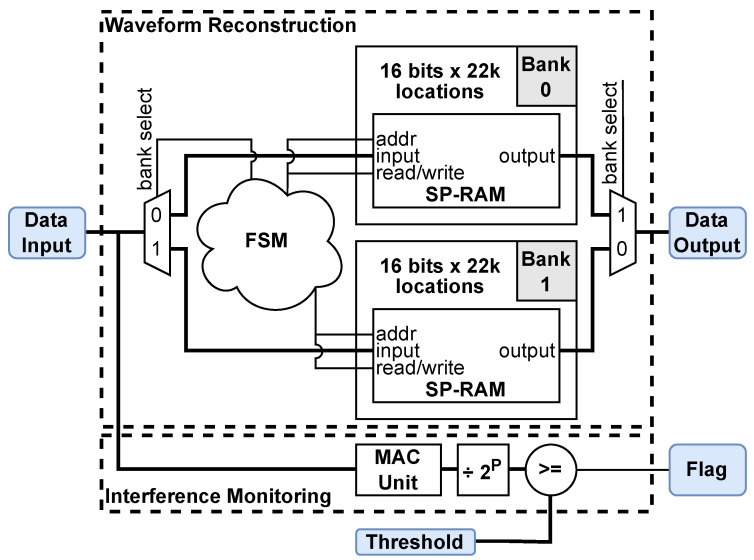
Block diagram of the hardware components within the FPGA used for waveform reconstruction and interference monitoring in one of the two channels. The two embedded processes run in parallel, and they are delineated with dashed-line boxes. The MAC unit is a multiplier-accumulator.

Figure 8 shows a block diagram of the hardware components within the FPGA used for parallelizing a single-channel waveform reconstruction and interference-monitoring preprocessing. In contrast with prior waveform reconstruction strategies [11,12,15,16,31], two single-port memory banks (SP-RAM in Figure 8) run in parallel. Each bank has 22,000 16-bit locations to store one fully sampled waveform. A local FSM controls the *bank select* signal to determine the bank in which the data are written (0 or 1). It also provides a memory address and read/write flag to the *addr* and *read/write* ports, respectively. While one bank is written to through the *input* port, the other is offloaded through the *output* port. This is the first parallelization approach, which achieves maximum throughput at the cost of a second memory resource.

The interleaving of the *K* sub-sampled waveforms is accomplished while writing to a bank by selecting the memory location (*addr* port in Figure 8) as addr=k+nK. This realizes the digitized signal in (4). When reading from a bank, the *addr* port is driven by a sequential counter from 0 to NK−1= 21,999. Since the number of locations written to equals those read from, and the *write* and *read* operations are both matched to the 200 MHz reference clock, the waveform reconstruction is 100% efficient (no data loss).

### 3.3. FPGA Signal Preprocessing

As shown in Figure 5, preprocessing within the FPGA performs *DC-offset removal*, *interference monitoring*, and *user-defined averaging*.

#### 3.3.1. DC-Offset Removal

A pre-determined DC offset is subtracted from all sub-samples. This value is ETSR-specific and is determined empirically. It is obtained by terminating the ETSR input ports with a 50 Ω load, resulting in the ETSR outputs being noise-only waveforms. For each channel, 1024 such waveforms are acquired and averaged, producing $x_{\mathrm{avg}}\left[i\right]$, i=0,…,NK−1. The DC offset is then computed as

(5) $$\bar{x}=\frac{1}{NK}\sum_{i=0}^{NK-1}x_{\mathrm{avg}}\left[i\right].$$

The DC offset values used here are 5.49 mV and 0.99 mV for RxA and RxB, respectively.

#### 3.3.2. Interference Monitoring

This preprocessing step mitigates the impact of EMI sources corrupting the received waveforms. It is the second parallel operation in the FPGA preprocessing stage. It runs in parallel with the waveform reconstruction (see Figure 8) and produces a flag when EMI is detected. This flag is submitted to the *user-defined averaging* module to discard the corrupted waveform, i.e., not including it in the average.

When the radar operates indoors, the strongest EMI is usually due to Wi-Fi transmissions. This poses a problem for the radar system, as the Wi-Fi packets, depending on their size and bit rate, spread over much longer periods (well over 100 μs) than the pulse period of 1 μs. This causes incoherent interference over the entire reconstructed waveform period, masking the back-scattered radar signal from a target. However, the UWB pulse is very short (∼4 ns, see Figure 1) compared to the 1 μs waveform. Thus, large portions of the reconstructed waveform carry only system noise (see Figure 4). As a result, the interference is easily detected within a predetermined portion of the waveform where the pulse does not exist. We define a time period at the beginning of waveform reconstruction and denote it as $T_{\mathrm{EMI}}=\tau_{k}+jT_{\mathrm{ADC}}$, where j=0,…,J−1, and J<N−1. $T_{\mathrm{EMI}}$ is user-defined by the number of samples. Here, we use KJ=4096, resulting in a temporal length of (J/N)μs≈186.2μs.

The detection of EMI employs the variance of the zero-mean signal within $T_{\mathrm{EMI}}$:

(6) $$\sigma^{2}=\frac{1}{KJ}\sum_{k=0}^{K-1}\sum_{j=0}^{J-1}{\left(x_{k}\left[j\right]-\bar{x}\right)}^{2}.$$

It is advantageous to have the sample length such that $KJ=2^{P}$ (here, P=12) since this allows for efficient fixed-point division. The FPGA hardware components implementing the variance computation (6) are the multiplier-accumulator along with the division by $2^{P}$ (see Figure 8). A user-defined threshold $\alpha^{2}$ is then used to determine whether a waveform reconstruction is corrupted by checking $\alpha^{2}\geq \sigma^{2}$.

The threshold α is dependent on the entire radar system. The threshold is determined by deploying the entire radar system in an electromagnetically quiet environment, such as an anechoic chamber. The system voltage noise standard deviation $\sigma_{v}$ is computed, as described in [10], for the $T_{\mathrm{EMI}}$ portion of the reconstructed waveform. Here, the EMI detection threshold is empirically chosen to be $\alpha =1.2\times \sigma_{v}$ or 1.21 mV. It is also important to emphasize that, due to the parallelization, each reconstructed pulse on the simultaneous channel collection can be inspected for EMI. This enables us to discard corrupted waveforms on both channels before averaging. If the EMI signals are below the interference-monitoring threshold, their impact is nonetheless mitigated by the averaging.

**Figure 9:**
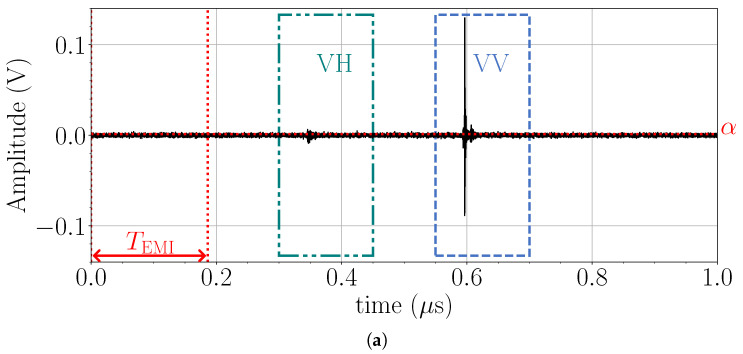

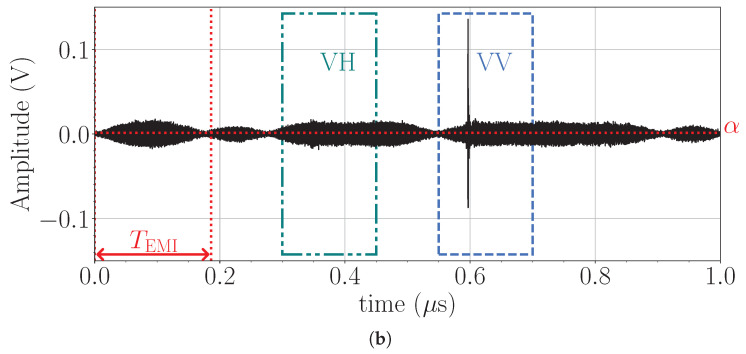
Illustration of reconstructed waveforms (**a**) with EMI suppression enabled, and (**b**) with a Wi-Fi burst corrupting the signal while EMI suppression is disabled. The dashed-dotted and dashed-line windows show the VH and VV responses, respectively. The vertical dotted lines show the interference-monitoring window $T_{\mathrm{EMI}}$. The horizontal dot line indicates the EMI threshold α.

Figure 9 illustrates an example of an indoor measurement of a dihedral corner reflector (aligned with the V-polarized Tx and Rx antennas). Only the waveform received by the V-polarized Rx antenna is shown, which contains the VV and VH signals. Figure 9a shows an example of a received waveform (without averaging) when EMI monitoring is enabled, whereas Figure 9b shows the same measurement scenario when it is disabled. Intense Wi-Fi communication is realized through a video call from a tablet. In Figure 9a, the VH and VV traces are clearly identified against the noise, while in Figure 9b, the weak VH signal is completely masked by the interference. We have determined empirically that this simple EMI mitigation strategy discards 3–6% of the reconstructed waveforms when the radar is deployed under the described scenario. 

#### 3.3.3. User-Defined Averaging

This process uses $2^{A}$, A=0,1,…,8, fully sampled waveforms to deliver a single cumulative average. Averaging improves the SNR for random uncorrelated noise by a factor of *A* [2], and cumulative averaging reduces the amount of offloaded data by a factor of $2^{A}$.

**Figure 10:**
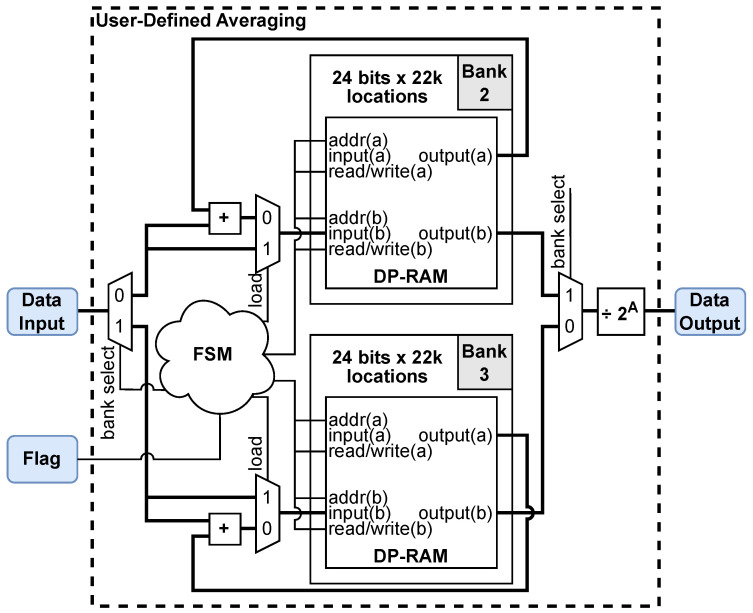
Block diagram illustrating the various hardware components in the FPGA used for user-defined averaging on a single channel.

A block diagram of the involved FPGA hardware components is shown in Figure 10. Similar to the *waveform reconstruction* module, two memory banks (Bank 2 and Bank 3) perform parallel processing, each containing 22,000 locations. Again, while one bank is written to, the other is offloaded from, controlled by the “bank select” signal. However, here, the bank locations are extended from 16 bits to 24 bits to support an accumulated sum of up to 256 ($2^{8}$) waveforms. The additional 8-bit width is needed to maintain precision and avoid overflow. Additionally, each bank is implemented with a dual-port RAM (DP-RAM) in the FPGA, allowing for two input/output port pairs: (a) and (b) in Figure 10. While the two port pairs share the same memory addresses, they allow for separate access to *write/read* operations. In particular, two output ports are needed in each dual-port RAM to implement averaging. The address ports, *addr* (a) and (b), for both *read* and *write* operations, are driven by sequential counters from 0 to NK−1= 21,999.

To obtain the $2^{A}$ average, the first incoming waveform is written to a memory address through the input (b), which is controlled by the “load” signal from the local FSM. Subsequent waveforms are accumulated by reading the last accumulated result from the output (a), adding it to the incoming waveform on input (b) and writing it to the same memory address. Once $2^{A}$ waveforms are accumulated, the bank is emptied on output (b), while the second bank begins the process again. The sum is divided by $2^{A}$ before offloading to the DDR3 memory.

The dual-bank design of the averaging module is 100% efficient with zero data loss. With A>0, the amount of data to be delivered to the external computer is also reduced. The data-reduction ratio resulting from the averaging ($DR_{\mathrm{av}}$) is the input-to-output sample ratio, i.e., $DR_{\mathrm{av}}\left(A\right)=2^{A}$. As explained later, this aids data offloading in matching the radar speed of one waveform every 110 μs.

### 3.4. CPU Signal Preprocessing and Data Offloading

#### 3.4.1. Trace Windowing

This task is performed by the CPU and is the last parallel processing step in the preprocessing pipeline. While the FPGA is preparing the next pair of reconstructed waveforms, the CPU is offloading the current pair of reconstructed waveforms. Here, the two prepared waveforms are segmented into four traces (VV, VH, HV, and HH), offloaded in this order. This allows the user to set the trace windows (depending on the anticipated range to the target), which can provide substantial data reduction. Table 2 presents some windowing cases used later for throughput analysis. The second column lists the number of samples in each trace. Since a full waveform contains 22,000 samples and two responses (VV and VH, or HV and HH), the maximum trace length is 11,000 samples. The last column shows the windowing data-reduction ratio $DR_{\mathrm{win}}$, i.e., the ratio of the maximum trace length to the actual one.

#### 3.4.2. Data Offloading

CPU-controlled data offloading is carried out via a 1 Gbps Ethernet link using the lightweight IP (LwIP) library [53]. Broadcast datagrams [57] are employed, which is important for eliminating the latency and throughput overhead inherent in the more common connection-based communication protocols [58]. The network spans only the radar and the external device, making it highly reliable, so most advantages of connection-based protocols are irrelevant. Note that broadcast datagrams are used in the connectionless user-datagram protocols. Each data packet sent includes a small header with sequence counters to enable data loss detection and proper data sequencing in the computer. The offloaded traces are then used for digital signal processing [59], system calibration [50], saving, or plotting in real time.

## 4. Results and Discussion

### 4.1. Firmware Throughput Efficiency, Data Reduction, and End-to-End Latency

Table 3 illustrates the calculation of the FPGA firmware data-throughput efficiency, as well as the respective data reduction for the trace-windowing case 5, as described in Table 2. The modules listed in the first column correspond to the signal-processing chain in Figure 5. The total efficiency is the product of all module efficiencies. When interference monitoring is disabled, a total efficiency of 98.2% is achieved. When interference monitoring is enabled, EMI-corrupt waveforms are discarded, thus reducing efficiency; however, this is not related to the firmware implementation. In summary, the proposed FPGA architecture can effectively match the radar measurement speed with minimal data loss.

The data reduction of each FPGA module is shown in the third column of Table 3. The *deserialization* and *DC-offset removal* modules have DR=1 since they do not reduce the amount of data. The *buffer* and *interference-monitoring* modules incur marginal data loss, hence the values just above 1. The most significant data reduction in the considered case 5 occurs in the *user-defined averaging* module due to averaging over eight waveforms and in the *trace-windowing* module due to windowing the traces to one-fifth of their full length. The total data reduction is the product of the DR values. As shown next, the minimum DR value necessary to offload the data without loss through the Ethernet link is about 40.

Finally, the FPGA end-to-end latency of the proposed FPGA design (provided that averaging is disabled) is measured at 224.07 μs since two reconstructed pulses (2×112 μs) are always in the pipeline, plus some registers (14 clocked at 5 ns) for data pipelining. Increasing the cumulative averaging by a factor of $2^{A}$ increases the latency by $2^{A-1}\times 112$ μs. Therefore, the worst-case end-to-end latency occurs for an average of $2^{8}$, which is ∼28.78 ms. This latency is reasonable and does not affect the application to real-time concealed weapon detection, as it remains below the worst-case acquisition time of 138 ms.

### 4.2. Data Offloading Throughput Analysis

To evaluate the data offloading throughput, each trace is tagged with a unique identification number by the CPU. This allows for counting and tracking the traces received by the computer. The analysis employs the cases listed in Table 2 along with varying averaging.

**Figure 11:**
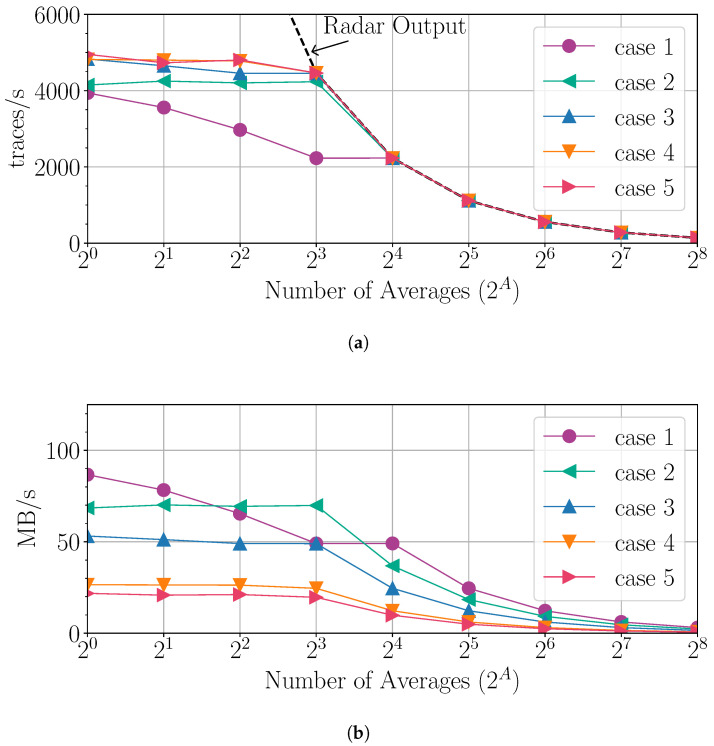
Data throughput analysis using 40,000 generated traces versus the number of averages: (**a**) traces per second received, (**b**) data throughput rate. The labels “case 1” to “case 5” refer to those described in Table 2.

Figure 11 presents the data offloading throughput analysis results using 40,000 generated traces versus the number of averages. Figure 11a shows the number of traces per second successfully offloaded to the computer. The dashed line shows the full number of traces generated by the radar (including the FPGA preprocessing chain), which is 98.2% of $4\times 9090/2^{A}$ traces/s. It is evident that the Ethernet offload saturates at about 4000 to 5000 traces/s depending on the trace length. When the number of averages is 8 or higher, there is no data loss, except in case 1 (full-length traces). For averaging below 8, the throughput improves as the traces become shorter, which is expected. For Ethernet offloading, it is advantageous to employ at least 8 averages, along with reducing the trace length by $DR_{\mathrm{win}}=1.33$ or more, since this fully utilizes the radar output and improves the SNR.

Figure 11b shows the data throughput rate. It can be observed that the theoretical network speed of 125 MB/s is never reached, which is due to the networking overhead. Also, sending longer traces (see cases 1 or 2) results in higher data rates, which can be explained by larger data payloads requiring less networking overhead per second. This insight can be used to further increase throughput by packing shorter traces into larger network packets (not pursued here).

The proposed design approach and architecture are scalable as technology evolves, making the ADC and Ethernet data rates faster. For instance, doubling the Ethernet speed while keeping the same ADC sampling rate allows for reducing the averaging, thus leading to higher throughput (as seen in Figure 11a). The enhanced throughput, currently constrained by today’s commonplace technology, would provide higher trace rates, thus improving detectability. Of course, higher-bandwidth solutions are currently available, such as multi-gigabit links via Ethernet, PCIe, and USB3/4 interfaces. Provided that the employed FPGA board can support these higher-bandwidth solutions, replacing the 1 Gbps Ethernet link is straightforward.

Conversely, increasing the ADC’s sampling rate would demand larger buffers and memory resources on the FPGA. For example, doubling the sampling rate or waveform period would require twice the number of memory locations per bank. The architecture would remain the same, provided that the FPGA has the memory resources to support the implementation. The FPGA throughput efficiency would not be affected by these increases due to the proposed parallel processing approach. Certainly, a larger ADC sampling rate would also lead to challenges with data offloading.

### 4.3. Comparison with Prior Works

As shown in this work, highlighting and addressing FPGA-based design inefficiencies and throughput capabilities are of great importance for achieving a loss-free, high-throughput, and capable radar system. This is often not investigated or reported in published works, leaving a gap in these systems’ performance metrics due to suboptimal solutions. For example, the system proposed in [12] was shown to produce a reconstructed image every 0.748 ms (1336 FPS), but the images are limited to a display refresh rate of 60 FPS. This effectively leads to 4.5% throughput efficiency. This result also includes image reconstruction, an application-specific solution not relevant to a generic receiver.

The designs proposed in [11,12,13,15,16,31] use a single memory bank for channel waveform reconstruction, averaging, and offloading. Data loss occurs in these designs due to the serialized signal-processing approach, but the extent of the loss is unclear. In contrast, we present a system that can achieve high efficiency and high-throughput capabilities far exceeding the user-defined output.

The reported (or extracted from available data) waveform reconstruction times for the designs proposed in [11,12,14] show that they require more than 144 pulse periods to reconstruct a 100 ns waveform. Our solution constructs a 1 μs waveform in 112 pulse periods, representing a significant improvement. It is important to note that making quantifiable comparisons is difficult due to the various ET sampling rates reported, ranging from 5 GSa/s to 100 GSa/s. The ET sampling rate employed directly impacts the number of pulse periods used, the FPGA memory management, and the temporal extent of the measurements. Comparisons are further inhibited by the lack of agreed-upon metrics for data loss and data throughput.

### 4.4. Experimental Validation Involving Walking People

Here, we demonstrate the ability of the proposed high-throughput radar system to capture thousands of responses from people walking across its field of view. Team members, one at a time with their consent, are asked to walk back and forth along the cross-range at a range distance of about 1 meter (similar to the scenario in Figure 2). The radar system with antennas is placed outside a semi-anechoic chamber with the antennas pointing toward the chamber. In one experiment, the person walks slowly, and in another, the person walks at a normal pace. A video camera captures the person’s motion, and each video frame is timestamped. This establishes the correspondence between the video capture and the radar measurement. The FPGA preprocessing employs an average of eight waveforms for trace-windowing case 5 (see Table 2). The measured traces are saved and plotted on a computer.

**Figure 12:**
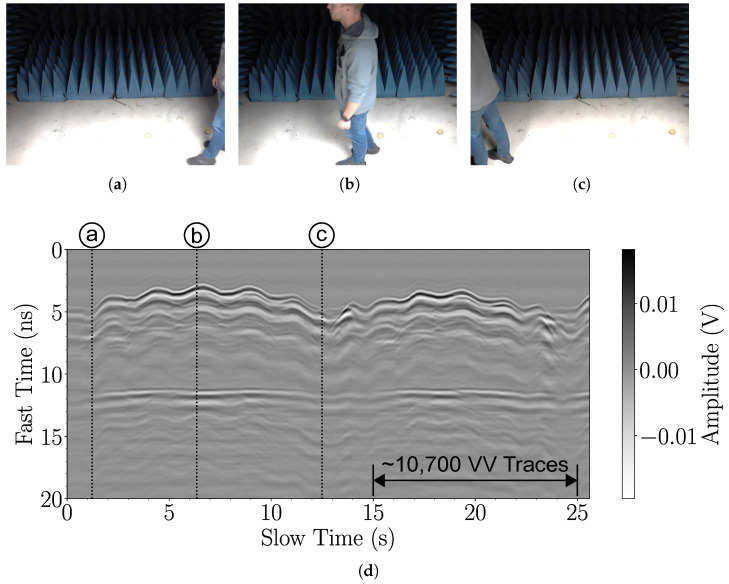
Measurements of a human walking slowly back and forth along the cross-range and at a range distance of about 1 meter from the antennas. The radar is positioned outside an open chamber with the antennas pointed toward the chamber. The person’s speed is estimated to be 0.22 m/s. Video frames show the positions of the person at (**a**) the start, (**b**) midway between the Tx and Rx antennas, and (**c**) the end after turning around. (**d**) The radargram shows the slow time versus the fast time of the VV radar response (background signal subtracted).

**Figure 13:**
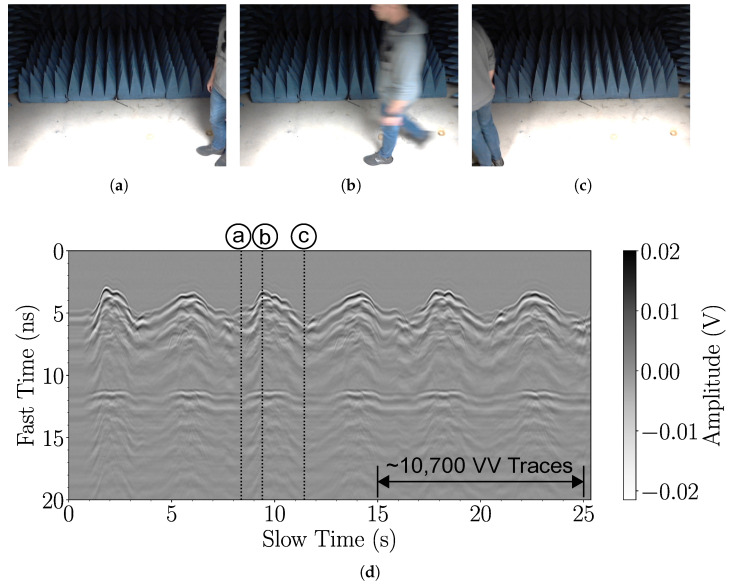
Measurements of a human walking normally back and forth along the cross-range and at a range distance of about 1 meter from the antennas. The setup is the same as that in Figure 12. The person’s speed is estimated to be 0.8 m/s. Video frames show the positions of the person at (**a**) the start, (**b**) midway between the Tx and Rx antennas, and (**c**) the end after turning around. (**d**) The radargram shows the slow time versus the fast time of the VV radar response (background signal subtracted).

Examples of these experiments are shown in Figure 12 and Figure 13. In Figure 12, the person walks slowly. The video captures the person’s position at the start, midway, and end, along with the corresponding peaks and dips in the radargram in Figure 12d. The radargram plots the slow time versus the fast time of 27,500 VV traces. The slow time corresponds to the time the pulse is received. The fast time corresponds to the temporal sample in the trace. Labels a, b, and c in Figure 12d correspond to the video frame timestamps in Figure 12a–c. A background response is obtained by averaging 27,500 traces without a person in the radar’s field of view. The background signal is subtracted to enhance the back-scattered response. Figure 13 is analogous to Figure 12 but for measurements with a person walking at a normal pace.

The radargrams in Figure 12 and Figure 13 clearly show the human response at around 3.5 ns, corresponding to a range of about 1 m. The peaks and troughs seen along the slow-time axis indicate the person’s movement. Peaks occur when the person is midway in the radar’s field of view, whereas troughs occur when the person reaches the end of the chamber and has to turn around. The person’s speed can be estimated using the timestamps of the radargram’s slow-time index. Measuring from trough to trough in Figure 12, the person’s speed is estimated to be 0.22 m/s. Likewise, in Figure 13, the person’s speed is estimated to be 0.8 m/s. In both figures, late-time (on the fast-time axis) ripples are observed that are only weakly dependent on the slow time. These are associated with multi-path responses.

## 5. Conclusions

A dual-channel FPGA-based architecture is proposed for a high-throughput UWB pulsed-radar system, which leverages parallel processing within the FPGA firmware. Parallel signal preprocessing is realized through a multi-memory bank layout, drastically improving the FPGA throughput efficiency and practically eliminating the data loss provided by the radar ETSR. It is shown that the FPGA-based signal preprocessing utilizes 98.2% of the high-speed data stream from the ETSR, effectively matching its speed. The realized radar system is equipped with an Ethernet link to a computer, and it is demonstrated that it can handle a total of about 4950 traces/s, with the limit dictated by the Ethernet capacity, not the radar system. Note that the FPGA-based design performs two simultaneous waveform reconstructions (each containing two responses) every 110μs, thus generating over 35,000 responses per second if averaging is disabled. Even with offloading limited to 4950 traces/s, the system still delivers 1238 traces/s in each radar response (VV, HH, VH, and HV). This remarkable speed makes the proposed radar system uniquely equipped for real-time radar applications targeting moving objects. Large datasets quickly become available for slowly moving objects such as people, making stochastic radar signal processing possible. Such processing provides powerful target detection and identification tools, which can successfully counteract environmental noise and radar clutter in emerging sensing and imaging UWB applications.

We also show how to leverage the speed of the proposed FPGA-based system to perform additional user-defined signal-preprocessing tasks besides ET waveform reconstruction, namely averaging, EMI suppression, and trace windowing. Averaging improves the SNR while reducing the amount of data to be offloaded to external devices. Trace windowing also greatly aids data reduction. EMI suppression allows for radar operation despite interference from wireless communications (e.g., Wi-Fi).

For the first time, FPGA firmware design strategies are delineated that are critical for improving the throughput efficiency of FPGA-controlled radar systems. The practical problem of data offloading is also discussed in the case of an Ethernet link since this offloading creates a bottleneck when additional signal processing is carried out on external devices. It is demonstrated that enabling data-reduction processing (averaging and trace windowing) within the FPGA firmware is critical for the optimal utilization of the high-speed radar output, i.e., achieving the best SNR for the given maximum data offloading rate.

## Figures and Tables

**Table 1:** UWB radar system parameters.

Parameters	Values
Lower Frequency, $f_{l}$	0.5 GHz
Upper Frequency, $f_{h}$	5.0 GHz
Bandwidth, *B*	4.5 GHz
Excitation	Monocycle Pulse
PRF, $f_{\mathrm{PRF}}$ (PRI, $T_{P}$)	1 MHz (1 μs)
Effective PRF, $f_{\mathrm{PRF},\mathrm{eff}}$ (Effective PRI, $T_{P,\mathrm{eff}}$)	0.5 MHz (0.5 μs)
Approximate Maximum Unambiguous Range, $R_{\max}$	75 m
Number of Transmitters	2
Number of Receivers	2
Reconstruction Method	ET Sampling
ET Sampling Rate, $f_{\mathrm{ET}}$ (Sampling Period, Δt)	20 GHz (50 ps)
ADC Sampling Rate, $f_{s}$ (Sampling Period, *T*)	200 MHz (5 ns)
ADC Resolution	16 bits
Sub-Sampled Waveforms per Reconstruction, *K*	110
Samples per Sub-Sampled Waveform, *N*	200
Number of Averages	$2^{A},\;A\leq 8$
Fully Sampled Waveform Reconstruction Time, $T_{\mathrm{Wfm}}$	110 μs

**Table 2:** Trace-windowing cases.

	Length in Sa/Trace	$DR_{\mathrm{win}}$
1	11,000	1
2	8250	1.33
3	5500	2
4	2750	4
5	2200	5

**Table 3:** FPGA firmware efficiency and data reduction when averaging over 8 waveforms for trace-windowing case 5.

Module	Efficiency (%)	Data Reduction, DR
Deserialization	100	1
Buffer	98.2	1.018
DC-Offset Removal	100	1
Waveform Reconstruction	100	1
Interference Monitoring	(94.0)	(1.06)
User-Defined Averaging	100	8
DMAC	100	1
Waveform Segmentation	100	5
	98.2 (92.3)	40.72 (43.16)

## Data Availability

Data is available upon request.

## Abbreviations

The following abbreviations are used in this manuscript:

**ADC**	Analog-to-digital converter
**AXI**	Advanced extensible interface
**BIST**	Built-in self-test
**CDC**	Clock-domain crossing
**CPU**	Central processing unit
**CWD**	Concealed weapon detection
**DMAC**	Direct memory access control
**EMI**	Electromagnetic interference
**ET**	Equivalent-time
**ETSR**	Equivalent-time sampling receiver
**FIFO**	First-in first-out
**FMC**	FPGA mezzanine card
**FPGA**	Field-programmable gate array
**FPS**	Frames per second
**FSM**	Finite state machine
**Gbps**	Gigabits per second
**GSa/s**	Gigasamples per second
**HPBW**	Half-power beamwidth
**LVDS**	Low-voltage differential signal
**LwIP**	Lightweight IP
**MAC**	Multiplier-accumulator
**PDC**	Programmable delay chip
**PLL**	Phase-Locked Loop
**PRF**	Pulse repetition frequency
**PRI**	Pulse repetition interval
**RF**	Radio frequency
**RT**	Real-time
**Rx**	Receiving
**SoC**	System-on-a-chip
**SNR**	Signal-to-noise ratio
**T&H**	Track-and-hold
**Tx**	Transmitting
**UWB**	Ultra-wideband
**Wfm/s**	Waveforms per second
